# Serial Cardiovascular Magnetic Resonance Studies Prior to and After mRNA-Based COVID-19 Booster Vaccination to Assess Booster-Associated Cardiac Effects

**DOI:** 10.3389/fcvm.2022.877183

**Published:** 2022-05-03

**Authors:** Claudia Meier, Dennis Korthals, Michael Bietenbeck, Bishwas Chamling, Stefanos Drakos, Volker Vehof, Philipp Stalling, Ali Yilmaz

**Affiliations:** Division of Cardiovascular Imaging, Department of Cardiology I, University Hospital Münster, Münster, Germany

**Keywords:** COVID-19, vaccination, CMR, MRI, myocarditis, t1-mapping, t2-mapping

## Abstract

**Background:**

mRNA-based COVID-19 vaccination is associated with rare but sometimes serious cases of acute peri-/myocarditis. It is still not well known whether a 3rd booster-vaccination is also associated with functional and/or structural changes regarding cardiac status. The aim of this study was to assess the possible occurrence of peri-/myocarditis in healthy volunteers and to analyze subclinical changes in functional and/or structural cardiac parameters following a mRNA-based booster-vaccination.

**Methods and Results:**

Healthy volunteers aged 18–50 years (*n* = 41; *m* = 23, *f* = 18) were enrolled for a CMR-based serial screening before and after 3rd booster-vaccination at a single center in Germany. Each study visit comprised a multi-parametric CMR scan, blood analyses with cardiac markers, markers of inflammation and SARS-CoV-2-IgG antibody titers, resting ECGs and a questionnaire regarding clinical symptoms. CMR examinations were performed before (median 3 days) and after (median 6 days) 3rd booster-vaccination. There was no significant change in cardiac parameters, CRP or D-dimer after vaccination, but a significant rise in the SARS-CoV-2-IgG titer (*p* < 0.001), with a significantly higher increase in females compared to males (*p* = 0.044). No changes regarding CMR parameters including global native T1- and T2-mapping values of the myocardium were observed. A single case of a vaccination-associated mild pericardial inflammation was detected by T2-weighted CMR images.

**Conclusion:**

There were no functional or structural changes in the myocardium after booster-vaccination in our cohort of 41 healthy subjects. However, subclinical pericarditis was observed in one case and could only be depicted by multiparametric CMR.

## Introduction

Without any doubt, COVID-19 vaccines are a blessing and prevented many millions of people world-wide from becoming either very ill or even dying due to a COVID-19 infection. Nevertheless, various reports showed that particularly mRNA-based COVID-19 vaccines are associated with rare but sometimes serious cases of acute peri-/myocarditis (1, 2). We still need to better understand why some people show cardiac adverse events following vaccination.

Magnetic resonance imaging (MRI) is the non-invasive gold standard in the diagnosis of myocardial inflammation (3). In this context, some impressive case reports presented severe myocardial damage on cardiovascular magnetic resonance imaging (CMR) even without functional impairment (4, 5), and the true number of COVID-19 vaccine-associated peri-/myocarditis may even be underreported since CMR is still not widely available.

So far, there are only limited data available regarding the frequency of vaccine-associated peri-/myocarditis following a 3rd booster vaccination for COVID-19 and regarding the value of CMR (6, 7). Hence, the aim of this prospective study was (a) to assess the possible occurrence of peri-/myocarditis following a mRNA-based booster vaccination in healthy volunteers and (b) to also analyze whether there are subclinical changes in functional and/or structural cardiac parameters possibly triggered by the preceding booster vaccination.

## Methods

Healthy volunteers aged 18–50 years were enrolled for a CMR-based serial screening before and after 3rd booster vaccination in the CMR-Center of University Hospital Muenster, Germany. Each study visit comprised a CMR scan, blood analyses with cardiac markers, markers of inflammation and SARS-CoV-2-IgG antibody titers, resting ECGs and a questionnaire regarding clinical symptoms. After their baseline examination, the study subjects received their 3rd booster dose of a mRNA-based COVID-19 vaccine with either mRNA-1273 (Moderna) or BNT162b2 (Pfizer-BioNTech) within 1–10 days. The follow-up examination was performed 4–10 days after booster vaccination.

Cardiovascular magnetic resonance imaging was performed on a 1.5 T-scanner (Philips Healthcare, Best, Netherlands) with a modified standard protocol used in clinical practice for suspected myocarditis (8). The protocol included high resolution cine, native T1- as well as T2-mapping, T2-STIR imaging and flow measurements. Contrast agent administration with additional late-gadolinium-enhancement (LGE) imaging was only intended if the native scan showed clear signs of active myocardial inflammation. Native T1- and T2- times were measured on three short axis views using pixelwise maps. All subjects gave their written informed consent to the study.

Skewed variables are expressed as median and interquartile range (IQR). Categorical variables are expressed as frequency with percentage. A *p*-value ≤ 0.05 was considered statistically significant.

## Results

Between November 2021 and January 2022, we prospectively examined 41 healthy, individuals with a median age of 35 years before (median 3 days) and after (median 6 days) their 3rd booster vaccination. The subjects (56% male) had no history of any cardiac disease or prior COVID-19 infection. There was one loss of follow up, because one participant experienced a COVID-19 infection in the interval between the third vaccination and the follow-up appointment. 30% of the subjects received mRNA-1273 (Moderna) and 70% received BNT162b2 (BioNTech) for booster (Table 1). No association between the subjective burden of symptoms and the respective increase in SARS CoV-2-IgG titer was observed.

**TABLE 1:** Subject characteristics.

Parameter			
Age, years	35 (31–38)		
Male/female, *n* (%)	23/18 (56%/44%)		
BMI, kg/m^2^	23.2 (22.2–24.4)		
Time between BL-CMR and booster vaccination, days	3.0 (1.3–6.0)		
Time between FUP-CMR and booster vaccination, days	(5.3–7.8)		
**Vaccine for 3rd vaccination**			
- BioNTech (BNT162b2)	28 (70%)		
- Moderna (mRNA-1273)	12 (30%)		
Allergies, *n* (%)	14 (34%)		

**Symptoms after 3rd vs. 2nd vaccination**	***n***	***n***	p*

- Local	34	22	**0.008**
- Fever	9	10	0.08
- Palpitations	4	1	0.27
- Chest pain	1	1	0.31
- Dyspnea	2	1	0.65
- Fatigue	22	24	0.67
- No symptoms	6	12	**0.011**

*If not stated otherwise all data are expressed as median (interquartile range).*

*Calculated for duration of symptoms not frequency among subjects. BMI, body mass index; BL-CMR, cardiac magnetic resonance at baseline; FUP-CMR, cardiac magnetic resonance at follow-up. The variables in bold show the significant correlations at a significance level of p.

There was no pathological elevation and no significant change in serum markers such as CK, CK-MB, high-sensitive troponin T, NT-proBNP, CRP or D-dimer before and after the 3rd booster vaccination (Table 2). As expected, there was a highly significant rise in the SARS-CoV-2-IgG titer (*p* < 0.001) in our study population. In addition, females showed a significantly higher increase in SARS-CoV-2-IgG titer (*p* = 0.044) compared to males.

**TABLE 2:** Cardiac magnetic resonance (CMR)-findings, laboratory, and ECG parameters.

Parameter	Pre booster	Post booster	*p*-Value
**➢ CMR findings**			
LV-EF,%	60 (55–63)	61 (57–64)	0.05
LV-EDVi, ml/m^2^	87 (80–95)	86 (90–94)	0.24
LV- GLS,%	−16.2 (−17.2 to −15.0)	−15.9 (−17.1 to −14.6)	0.75
RV-EF,%	55 (50–59)	54 (51–57)	0.85
RV-EDVi, ml/m^2^	92 (83–102)	91 (80–100)	0.48
LV-mass, g/m^2^	47 (42–54)	47 (42–54)	0.61
Global native T1, ms	988 (964–1,011)	983 (970–1,024)	0.90
No. of segments with elevated T1 Mapping value > 1,060 ms, *n* (%)	0 (0%)	0 (0%)	
Global T2, ms	50 (49–51)	50 (49–51)	0.40
No. of segments with elevated T2 Mapping value > 59 ms, *n* (%)	0 (0%)	0 (0%)	
Presence of edema in T2 weighted images,			
- myocardial, *n* (%)	0 (0%)	0 (0%)	
- pericardial, *n* (%)	2 (1.6%)	3 (2.6%)	
**➢ Biochemistry marker**			
CK, U/l	113 (83–187)	99 (78–133)	0.07
CK-MB, U/l	14 (12–16)	14 (12–17)	0.50
Troponin T, ng/l	3.0 (3.0–4.7)	3.0 (3.0–4.3)	0.59
NT-pro-BNP, pg/ml	30 (13–58)	21 (11–52)	0.26
D-dimer, mg/l	0.27 (0.27–0.27)	0.27 (0.27–0.28)	0.39
CRP, mg/dl	0.5 (0.5–0.5)	0.5 (0.5–0.5)	0.32
SARS CoV-2-IgG, AU/ml	1,319 (681–1,788)	16,077 (10,312–32,540)	**<0.001**
SARS CoV-2-IgG in male, AU/ml	1,807 (601–2,485)	15,643 (9,129–19,650)	**<0.001**
SARS CoV-2-IgG in female, AU/ml	2,076 (691–1,717)	24,271 (11,092–40,000)	**<0.001**
Δ SARS CoV-2-IgG, AU/ml	*Male*	*Female*	
	13,388 (8,873–15,927)	20,640 (9,332–38,637)	**0.044**
**➢ ECG parameters**			
Heart rate, bpm	67 (60–75)	71 (64–78)	0.06
**ST-elevation**			
- minor < 0,1 mV, *n*	14 (34%)	12 (30%)	
- significant ≥ 0,1 mV, *n*	0 (0%)	0 (0%)	
ST-depression ≥ -0,1 mV, *n*	0 (0%)	0 (0%)	

*If not stated otherwise all data are expressed as median (interquartile range); All biochemistry marker (with exception of SARS CoV-2-IgG) are in normal range of values.*

*LV-EF, left ventricular ejection fraction; LV-EDVi, indexed left ventricular end-diastolic volume; LV-GLS, left ventricular global longitudinal stain; RV-EF, right ventricular ejection fraction; RV-EDVi, indexed right ventricular end-diastolic volume; CK, creatinine kinase; CK-MB, creatinine kinase isoenzyme MB; NT-pro BNP, N-terminal -pro brain natriuretic peptide; CRP, C-reactive protein; SARS CoV-2-IgG, anti-severe acute respiratory syndrome coronavirus 2 -immunoglobulin G. Δp – significance between the changes among IgG rise in male and female. p < 0.05 is considered as significant. The variables in bold show the significant correlations at a significance level of p.*

**FIGURE 1:**
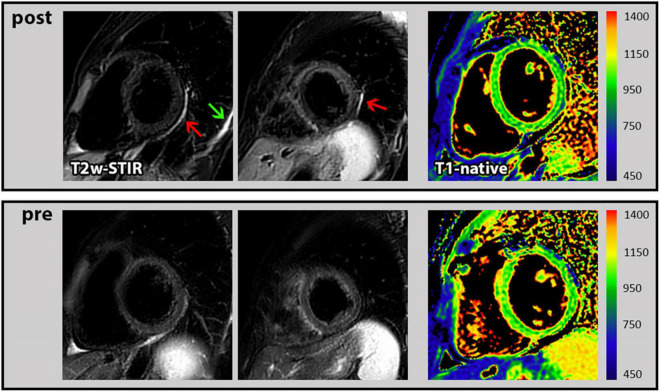
Cardiac magnetic resonance (CMR) images of pericarditis. *First row*: T2-STIR-weighted short-axis images with the occurrence of pericardial hyperintensity as indication for edema/mild pericardial inflammation (red arrow) and a new pleural effusion (green arrow) following the 3rd COVID-19 vaccination. In addition, corresponding T1 mapping without signs of myocardial impairment. *Second row*: Corresponding images at baseline (prior to 3rd COVID-19 vaccination) from the same subject without any pathological findings.

In general, the assessment of both functional as well as structural CMR parameters showed highly consistent and reproducible values when the respective CMR parameters measured before and after the booster vaccination were compared. In particular, there was no change in biventricular function and volumes, in global longitudinal strain and in myocardial mass (Table 2). Moreover, the global native T1- and T2-mapping values remained unchanged (988 vs. 983 ms in T1 and each 50 ms in T2). There was one female who demonstrated a new “pericardial” T2-STIR-weighted hyperintensity in the basal to midventricular inferolateral pericardium and also a new pleural effusion (Figure 1). In the absence of any symptoms or signs of other diseases, we interpreted these findings as a vaccination-associated form of very mild pericardial inflammation. There was no known clinical characteristic or laboratory parameter that could provide a predisposition to pericarditis in this case.

## Discussion

Although the pivotal approval studies, sponsored by the respective pharmaceutical companies, did not show an increased risk of myocarditis following COVID-19 vaccination (9, 10), today there is no doubt that mRNA-based COVID-19 vaccination can cause peri-/myocarditis particularly in young males (1, 11, 12). It has also been shown that the risk of myocarditis is predominantly increased after the second vaccination dose. Assuming an autoimmune-mediated process, it is still unknown whether a 3rd booster vaccination is also associated with a non-neglectable risk of peri-/myocarditis.

To the best of our knowledge, our present study is the first one that used multi-parametric serial CMR studies prior to and after mRNA-based COVID-19 booster vaccination to carefully assess potential booster-associated cardiac effects. Our major findings can be summarized as follows:

First, the present data show that no relevant myocardial changes could be observed by CMR following the 3rd booster vaccination. Our data support current recommendations regarding booster vaccinations that should not be withheld for fear of adverse cardiac events in healthy subjects aged <50 years (considering that there is no vaccination with mRNA-1273 (Moderna) in subjects <30 years since cases of peri-/myocarditis were more frequently observed after Moderna vaccination in this age group).

Second, subclinical pericarditis was observed in 1 out of 40 subjects following a 3rd booster vaccination whereas no cases of myocarditis were observed in the present study. Importantly, multi-parametric CMR imaging was the only diagnostic modality that allowed to depict such a mild pattern of pericardial inflammation. In line with this findings, a large descriptive study, based on reports to the VEARS (Vaccine Adverse Event Reporting System) reported only 17% of abnormal findings based on echocardiograms (in the cohort of myocarditis patients younger than 30 years), but abnormalities were reported in >70% by CMR (1).

Today, CMR is well-known and robust modality for the non-invasive diagnosis of myocarditis that does not only detect regional dysfunction, but allows also to depict edema and other subtle structural changes based on elevated T1- and T2-mapping values and/or characteristic patterns of LGE (13, 14). Therefore, the Lake Louise criteria for CMR-based diagnosis of myocardial inflammation were already established in 2009 and updated in 2018 (15, 16). Since the diagnosis of vaccine-associated myocarditis is important for symptom management, for exercise recommendations, for further cardiomyopathy monitoring and future (e.g., booster) vaccination decisions (3), physicians should be aware of the potential of multi-parametric CMR.

Last but not least, our present data clearly show that a 3rd booster vaccination leads to a substantial increase in the SARS CoV-2-IgG antibody titer – and interestingly to a higher increase in females compared to males. Hence, gender-based differences should be evaluated more carefully in future studies.

## Conclusion

The present serial CMR data support current recommendations regarding the safety of 3rd booster vaccinations since no functional or subtle structural changes were observed in the myocardium – as long as current vaccination recommendations are followed. However, subclinical pericarditis was observed in one case and could only be depicted by multiparametric CMR.

## Data Availability Statement

The original contributions presented in the study are included in the article/supplementary material, further inquiries can be directed to the corresponding author.

## Ethics Statement

Ethical approval was not provided for this study on human participants because written consent of participants were obtained and no identifiable images or data are presented in the study. The patients/participants provided their written informed consent to participate in this study.

## Author Contributions

CM devised the project, performed CMR examinations, performed major analyses of the CMR data, and drafted the manuscript. DK performed the CMR analyses and participated in the interpretation of results. MB participated in the design of the study, performed CMR examinations, and statistical analyses. BC participated in the design of the study, CMR exams, and critically reviewed the manuscript. SD participated in the CMR exams and in the analysis of the CMR data. VV and PS performed CMR examinations and participated in the analysis of clinical data. AY provided the main conceptual idea, supervised the work, provided critical feedback and helped shape the research, analysis, and manuscript. All authors contributed to the article and approved the submitted version.

## Conflict of Interest

The authors declare that the research was conducted in the absence of any commercial or financial relationships that could be construed as a potential conflict of interest.

## Publisher’s Note

All claims expressed in this article are solely those of the authors and do not necessarily represent those of their affiliated organizations, or those of the publisher, the editors and the reviewers. Any product that may be evaluated in this article, or claim that may be made by its manufacturer, is not guaranteed or endorsed by the publisher.

## References

[1] Oster ME Shay DK Su JR Gee J Creech CB Broder KR Myocarditis cases reported after mRNA-Based COVID-19 vaccination in the US From December 2020 to August 2021. *JAMA.* (2022) 327:331–40. 10.1001/jama.2021.24110 35076665 PMC8790664

[2] Albert E Aurigemma G Saucedo J Gerson DS. Myocarditis following COVID-19 vaccination. *Radiol Case Rep.* (2021) 16:2142–5. 34025885 10.1016/j.radcr.2021.05.033 PMC8130498

[3] Tschöpe C Ammirati E Bozkurt B Caforio ALP Cooper LT Felix SB Myocarditis and inflammatory cardiomyopathy: current evidence and future directions. *Nat Rev Cardiol.* (2021) 18:169–93. 10.1038/s41569-020-00435-x 33046850 PMC7548534

[4] Chamling B Vehof V Drakos S Weil M Stalling P Vahlhaus C Occurrence of acute infarct-like myocarditis following COVID-19 vaccination: just an accidental co-incidence or rather vaccination-associated autoimmune myocarditis? *Clin Res Cardiol.* (2021) 110:1850–4. 10.1007/s00392-021-01916-w 34333695 PMC8325525

[5] Kim HW Jenista ER Wendell DC Azevedo CF Campbell MJ Darty SN Patients with acute myocarditis following mRNA COVID-19 vaccination. *JAMA Cardiol.* (2021) 6:1196–201. 10.1001/jamacardio.2021.2828 34185046 PMC8243258

[6] Atmar RL Lyke KE Deming ME Jackson LA Branche AR El Sahly HM Homologous and heterologous Covid-19 booster vaccinations. *N Engl J Med.* (2022) 386:1046–57. 10.1056/NEJMoa2116414 35081293 PMC8820244

[7] Shiyovich A Witberg G Aviv Y Kornowski R Hamdan A. A case series of myocarditis following third (Booster) Dose of COVID-19 vaccination: magnetic resonance imaging study. *Front Cardiovasc Med.* (2022) 9:839090. 10.3389/fcvm.2022.839090 35310989 PMC8930918

[8] Kramer CM Barkhausen J Bucciarelli-Ducci C Flamm SD Kim RJ Nagel E. Standardized cardiovascular magnetic resonance imaging (CMR) protocols: 2020 update. *J Cardiovasc Magn Reson.* (2020) 22:17. 10.1186/s12968-020-00607-1 32089132 PMC7038611

[9] Food and Drug Administration [FDA], Center for Biologics Evaluation and Researc [CBER]. *Vaccines and Related Biological Products Advisory Committee December 17, 2020 Meeting Briefing Document - FDA, CBER, Biologics, COVID-19 FDA Briefing Document Moderna COVID-19 Vaccine: Published online December 17, 2020.* Silver Spring, MD: Food and Drug Administration (2020).

[10] Covid PB. *Pfizer-Biontech COVID-19 Vaccine (BNT162, PF-07302048) Vaccines and Related Biological Products Advisory Committee Briefing Document.* Silver Spring, MD: Food and Drug Administration (2020). p. 92

[11] Montgomery J Ryan M Engler R Hoffman D McClenathan B Collins L Myocarditis following immunization with mRNA COVID-19 vaccines in members of the US Military. *JAMA Cardiol.* (2021) 6:1202–6. 10.1001/jamacardio.2021.2833 34185045 PMC8243257

[12] Castiello T Georgiopoulos G Finocchiaro G Claudia M Gianatti A Delialis D COVID-19 and myocarditis: a systematic review and overview of current challenges. *Heart Fail Rev.* (2022) 27:251–61. 10.1007/s10741-021-10087-9 33761041 PMC7988375

[13] Yilmaz A Ferreira V Klingel K Kandolf R Neubauer S Sechtem U. Role of cardiovascular magnetic resonance imaging (CMR) in the diagnosis of acute and chronic myocarditis. *Heart Fail Rev.* (2013) 18:747–60. 10.1007/s10741-012-9356-5 23076423

[14] McDonagh TA Metra M Adamo M Gardner RS Baumbach A Böhm M 2021 ESC Guidelines for the diagnosis and treatment of acute and chronic heart failure. *Eur Heart J.* (2021) 42:3599–726. 34447992 10.1093/eurheartj/ehab368

[15] Friedrich MG Sechtem U Schulz-Menger J Holmvang G Alakija P Cooper LT Cardiovascular magnetic resonance in myocarditis: a JACC White Paper. *J Am Coll Cardiol.* (2009) 53:1475–87. 10.1016/j.jacc.2009.02.007 19389557 PMC2743893

[16] Ferreira VM Schulz-Menger J Holmvang G Kramer CM Carbone I Sechtem U Cardiovascular magnetic resonance in nonischemic myocardial inflammation: expert recommendations. *J Am Coll Cardiol.* (2018) 72:3158–76. 10.1016/j.jacc.2018.09.072 30545455

